# The Molecular Mechanisms of Portal Vein Thrombosis in Hepatocellular Carcinoma

**DOI:** 10.3390/cancers16193247

**Published:** 2024-09-24

**Authors:** Linda Galasso, Lucia Cerrito, Fabrizio Termite, Irene Mignini, Giorgio Esposto, Raffaele Borriello, Maria Elena Ainora, Antonio Gasbarrini, Maria Assunta Zocco

**Affiliations:** 1 Department of Internal Medicine and Gastroenterology, Fondazione Policlinico Universitario Agostino Gemelli IRCCS, Catholic University of Rome, 00168 Rome, Italy; linda.galasso@guest.policlinicogemelli.it (L.G.); mariaelena.ainora@policlinicogemelli.it (M.E.A.);; 2 CEMAD Digestive Disease Center, Fondazione Policlinico Universitario Agostino Gemelli IRCCS, Catholic University of Rome, 00168 Rome, Italy

**Keywords:** hepatocellular carcinoma, thrombosis, cirrhosis, endothelial disfunction

## Abstract

**Simple Summary:**

Hepatocellular carcinoma (HCC) associated with portal vein thrombosis (PVT) represents an advanced stage of the tumor, with a subsequently poor prognosis and reduced therapeutic perspectives. These patients could present severe complications such as ascites, metastases, an increase in portal hypertension and potentially fatal gastrointestinal bleeding. The intricate molecular patterns at the basis of the development of TVP could also represent possible biomarkers in the early detection of patients at high risk of PVT.

**Abstract:**

Hepatocellular carcinoma (HCC) represents the sixth most diagnosed cancer worldwide and is the second leading cause of cancer-related death in the world. The association of HCC and portal vein thrombosis (PVT) represents an advanced stage of the tumor. PVT has a prevalence of about 25–50% in HCC, determining poor prognosis and a remarkable reduction in therapeutic perspectives in these patients, leading to severe complications such as ascites, metastasis, an increase in portal hypertension and potentially fatal gastrointestinal bleeding. The aim of this review is to evaluate the molecular mechanisms that are at the basis of PVT development, trying to evaluate possible strategies in the early detection of patients at high risk of PVT.

## 1. Introduction

Portal vein thrombosis (PVT) is rare, occurring in 2–4 per 100,000 individuals without chronic liver disease [1]. Its incidence is much higher among those with cirrhosis, often indicating the severity of the disease [2]. In compensated cirrhosis cases, PVT is found in less than 10% of patients [3,4]. This prevalence rises to 17% in decompensated cirrhosis and can reach up to 40% in individuals with hepatocellular carcinoma (HCC) [5,6,7,8]. Furthermore, the presence of portal thrombosis, given the clinical symptoms it causes, often allows for the diagnosis of HCC itself [9]. HCC is the sixth most frequently diagnosed tumor and is generally the final stage of cirrhotic liver [10]. Diagnosing portal vein thrombosis in the context of HCC indicates a reduced life expectancy, as the disease progression in terms of ascites, metastases, and portal hypertension with associated gastrointestinal bleeding diminishes therapeutic prospects [11].

In the presence of HCC, it is possible to diagnose both portal vein thrombosis itself and portal vein thrombosis due to direct tumor invasion [9].

Differentiating between portal vein thrombosis caused by tumor invasion and bland portal vein thrombosis is crucial, as it has a major impact on clinical treatment decisions, especially regarding eligibility for liver transplantation. Additionally, the diagnosis of bland portal vein thrombosis poses a considerable challenge to performing locoregional procedures, such as transarterial chemoembolization (TACE) and radioembolization for HCC nodules, as it can diminish the effectiveness of these therapies [11,12].

Conversely, diagnosing portal vein tumor thrombosis (PVTT) can promote the use of locoregional therapies. Studies have shown that combining systemic treatments like Sorafenib with locoregional methods often produces better outcomes than systemic therapy alone [13,14].

Sherman CB et al. created the A-VENA algorithm to distinguish between tumor-related and bland PVT. This method combines ultrasound results with a laboratory marker, particularly alpha-fetoprotein levels with a 100% sensitivity and 94% specificity [15]. On color Doppler, PVTT linked to HCC is identified by enhancement or Doppler flow within the thrombus. Rossi S. et al. revealed that contrast-enhanced ultrasound (CEUS) outperforms CT in differentiating PVTT from bland thrombosis, perfect sensitivity and specificity [16].

The pathophysiology of PVT is complex, encompassing various molecular pathways and mechanisms like Virchow’s triad (changes in blood flow, endothelial dysfunction and hypercoagulability). Additionally, it involves inflammatory pathways, portal hypertension, genetic and epigenetic factors, extracellular vesicles (EVs), impaired fibrinolysis, and interactions within the gut–liver axis.

Our goal is to clarify each of these mechanisms, focusing on their molecular aspects that contribute to the development of portal vein thrombosis in cirrhotic patients with HCC.

## 2. Endothelial Dysfunction and Portal Vein Thrombosis in Cirrhotic Patients with a Focus on HCC: Molecular Mechanisms and Pathophysiology

The liver obtains the majority of its blood from the portal vein (80–90%), with a smaller portion supplied by the hepatic artery (10–20%). This blood passes through the hepatic sinusoids, which are capillaries with a fenestrated endothelial lining, and then drains into the suprahepatic veins. Sinusoidal endothelial cells (LSECs) are distinct due to their fenestrations and lack of a basement membrane under typical conditions. This specialized structure enhances nutrient delivery to hepatocytes. LSECs play a vital role in controlling sinusoidal blood flow by secreting vasoactive compounds that influence hepatic stellate cells (HSCs). For instance, when endothelin 1 (ET-1) binds to endothelin A (ETa) receptors on HSCs, it causes contraction, whereas nitric oxide (NO) promotes relaxation [17]. Liu S et al. further showed that ET-1 binding to endothelin B receptors (ETBR) leads to vasodilation through the phosphorylation of Akt and endothelial nitric oxide synthase (eNOS) via G-protein-coupled receptor signaling in a normal liver [18]. In cirrhotic livers, the functionality of LSECs is compromised, marked by the loss of fenestrations and the development of a basement membrane, a process called capillarization [19,20]. This change in hepatic structure increases portal pressure, leading to further endothelial damage and perpetuating a detrimental cycle. In cirrhotic livers, NO production decreases due to multiple factors. A significant factor is the upregulated expression of G protein-coupled receptor kinase 2 (GRK2) by LSECs, which hinders G-protein-coupled receptor signaling, thereby impairing Akt phosphorylation and diminishing NO synthesis [21].

Moreover, the inflammatory environment and oxidative stress associated with cirrhosis-induced structural changes impair the activity of eNOS in LSECs. This issue is due to greater interactions between eNOS and caveolin-1, which inhibit eNOS, combined with fewer interactions with ETB receptors that typically activate eNOS [22]. Consequently, NO production is reduced, increasing vascular resistance in the intrahepatic circulation.

The oxidative damage also disrupts the endothelial barrier, reduces hepatocyte oxygenation, resulting in cellular apoptosis, expression of damage-associated molecular patterns (DAMPs), and increased vascular permeability, and promotes inflammation and thrombosis. In this context, vascular endothelial growth factor (VEGF) is produced, which induces signaling cascades to activate Akt and subsequently eNOS through phosphorylation at Serine 1177 (human) [23]. As demonstrated by Abraldes JG et al., and Fernandez M et al., blocking VEGF receptor 2 can ameliorate arterial vasodilation in cirrhosis with portal hypertension [23,24]. Additionally, an increased production of the COX-1-derived vasoconstrictor prostanoid Thromboxane A2 (TXA2) by LSECs exemplifies endothelial dysfunction in cirrhosis [25].

In cirrhosis, the inflammatory system features increased concentrations of tumor necrosis factor-alpha (TNF-α), interleukin-6 (IL-6), and various other pro-inflammatory cytokines, including IFN-γ, TGF-β, interleukin-17 (IL-17), interleukin-9 (IL-9), interleukin-1β (IL-1β), and chemokines like CXCL8/CXCL1 and CCL2, all of which contribute to endothelial damage [26].

Inflammatory cytokines induce neutrophil activation and initiate the NFκB pathway, leading to heightened P-selectin expression that modulates endothelial–platelet adhesion. This pathway also upregulates E-selectin and interleukin 8 (IL-8) expression, thereby amplifying and sustaining the inflammatory response. These molecules facilitate leukocyte attachment to endothelial cells, promoting inflammation and platelet aggregation, thereby supporting the formation of thrombi [27,28,29]. Specifically, leukocytes are recruited to sites of thrombo-inflammation through direct interactions with activated endothelium.

Upon activation, there is an increase in P-selectin expression, which facilitates the adherence and rolling of neutrophils by binding to P-selectin glycoprotein ligand-1 (PSGL-1). Additionally, heightened levels of VCAM-1 (vascular cell adhesion molecule-1) and ICAM-1 (intercellular adhesion molecule-1) promote greater neutrophil attachment to the endothelial surface [30]. These interactions also contribute to the activation of HSCs, leading to increased cellular fibrosis and consequently, portal hypertension—an established risk factor for thrombosis [26].

Considerable research has been directed towards understanding the function of neutrophil extracellular traps (NETs) in thrombosis, released in inflammatory settings. Specifically, these traps contribute significantly to the thrombotic process by releasing enzymes like neutrophil elastase (NE), cathepsin-G, and myeloperoxidase (MPO), which activate factor XII [31].

Moreover, NETs contain tissue factor (TF), which contributes to platelet recruitment and activation through interactions with von Willebrand factor (VWF) [31]. Recently, Xu X et al. examined the role of NETs in assessing the thrombotic risk in cirrhotic patients undergoing non-selective β-blockers (NSBB) therapy, such as propranolol [32]. Furthermore, NETs can stimulate thrombin production, activate endothelial cells, and contribute to thrombus formation via a platelet-dependent mechanism involving Toll-like receptors 2 (TLR2) and Toll-like receptors 4 (TLR4) [33].

As previously mentioned, DAMPs play a crucial role in the activation of endothelial cells and the manifestation of thrombo-inflammatory conditions. Specifically, High-Mobility Group Box 1 (HMGB1), which is secreted by stimulated macrophages and activated platelets, is a prototypical DAMP. As shown by Pilard M. et al., in a mouse model of venous thromboembolism (VTE), HMGB1 rapidly accumulates on the endothelial surface and increases over time, correlating with the formation of platelet-leukocyte aggregates within the first hour of thrombosis induction [30]. Inhibiting HMGB1 pharmacologically has demonstrated efficacy in reducing the size of blood clots [34,35]. Furthermore, research conducted by Tadie J.M. et al. and Puricelli C. et al. highlights platelets as the main producers of HMGB1, thereby promoting the creation of NETs [36,37]. Deleting HMGB1 specifically in platelets in this context decreases both NET formation and blood clot size [38]. Furthermore, HMGB1 enhances the secretion of cytokines, notably Interleukin-8 (IL-8), which is associated with increased expression of ICAM-1 and VCAM-1. This promotes the recruitment and adhesion of neutrophils to endothelial cells [32].

**Figure 1:**
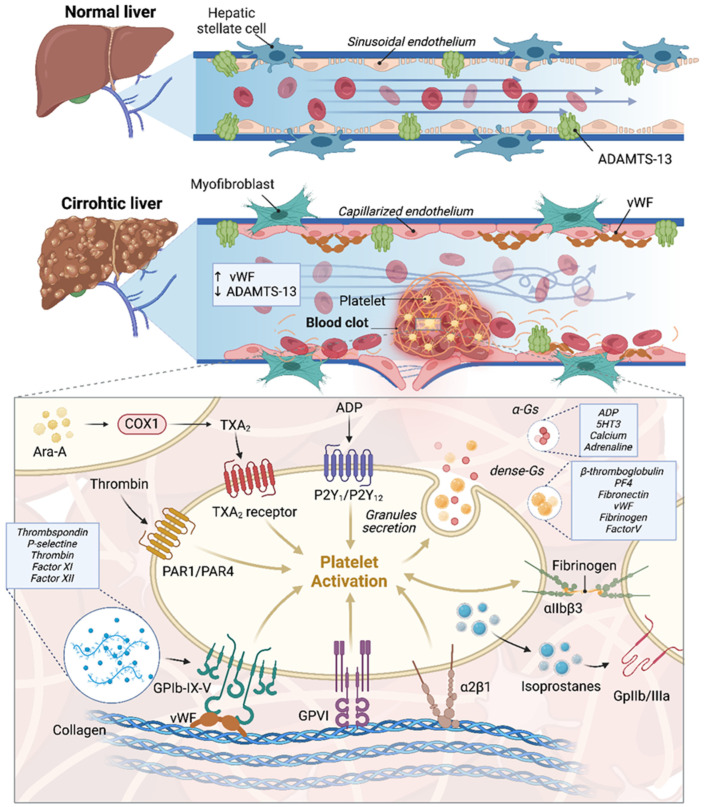
Differences between a sinusoid in a cirrhotic liver and one in a non-cirrhotic liver and molecular mechanisms of endothelial disfunction in the origin of portal vein thrombosis. This image has been generated with Biorender.com (https://biorender.com)—accessed on 14 August 2024.

In Figure 1, the significant differences between a sinusoid in a cirrhotic liver and one in a non-cirrhotic liver are illustrated.

If cirrhosis already induces well-established endothelial damage, it is clear how hepatocellular carcinoma (HCC), emerging in a pro-thrombotic environment, can further exacerbate this process through specific tumor factors. Indeed, inflammation plays a predominant role in the HCC microenvironment [39]. Particularly, HCC cells themselves secrete cytokines such as IL-6 and TNF-α, thereby increasing their concentration at the endothelial level and causing damage that renders endothelial cells lining the portal vein more susceptible to thrombosis [39]. Furthermore, within the tumor microenvironment of HCC, there is not only extracellular matrix remodeling but also endothelial-to-mesenchymal transition (EMT) in vascular endothelial cells, characterized by decreased E-cadherin (an epithelial marker) and increased N-cadherin (a mesenchymal marker) [40]. This transition is mediated by Cyclin G1 through the PI3K/Akt/GSK-3/Snail pathway [41]. The finding of high Cyclin G1 expression in portal vein thrombosis suggests a link between HCC-induced EMT and portal thrombosis formation [40].

The following paragraph elaborates on how endothelial dysfunction contributes to platelet aggregation. As this topic intersects with both endothelial dysfunction and the coagulation mechanism, we have chosen to explain it in detail in the next section.

## 3. Molecular Insights into Coagulopathy and Thrombosis Mechanisms in Cirrhotic Patients with a Focus on HCC

The current understanding of coagulopathy in cirrhotic individuals emphasizes a delicate balance of hemostasis involving both pro-hemostatic and anti-hemostatic factors [42].

Platelets are crucial in primary hemostasis for the formation of the initial hemostatic clot [Figure 1]. The adhesion of platelets to damaged vessel walls begins with the interaction of exposed collagen with platelet surface receptors GPVI and integrin α2β1, as well as the attachment of VWF to the GPIb-IX-V complex on the platelet surface [43].

This complex binds to several platelet ligands such as thrombospondin, P-selectin, leukocytes, thrombin, factor XI, and factor XII [42]. Thrombin, generated through the coagulation cascade, significantly activates human platelets by attaching to two distinct surface protease activated receptors, PAR-1 and PAR-4 [44]. Platelets exhibit a self-amplifying pro-thrombotic action. Upon activation, platelets release adenosine diphosphate (ADP) from dense granules, which binds to P2Y1 and P2Y12 receptors, and serotonin (5HT), which binds to 5HT2A receptors, as well as calcium and adrenaline, all released from dense granules. Additionally, activated platelets release growth factors such as beta-thromboglobulin, PF4, fibronectin, vWF, fibrinogen, and Factor V from alpha granules, contributing to thrombus formation [45].

Platelets generate TXA2 through the Cyclooxygenase 1 (COX1)-dependent pathway, enhancing thrombus formation [45]. Fibrinogen and VWF binding to activated integrin αIIbβ3 facilitate platelet-to-platelet aggregation [46].

Cirrhotic patients often present with thrombocytopenia, indicating an increased susceptibility to bleeding compared to those without cirrhosis. However, patients with cirrhosis also manifest platelet dysfunction [47]. Platelets in these individuals show elevated levels of isoprostanes, which activate the glycoprotein IIb/IIIa receptor, thereby intensifying platelet aggregation. Moreover, platelets release transforming growth factor beta (TGF-β), which in vitro studies indicate can upregulate endothelial vWF, thrombomodulin (TM), ICAM-1, and VEGF [48]. This contributes to endothelial dysfunction and heightens the thrombotic risk. Furthermore, in cirrhosis, thrombocytopenia is counterbalanced by significantly elevated levels of vWF and concurrently low levels of ADAMTS-13 [49]. In cirrhotic livers, LSECs play a key role in the storage and synthesis of vWF and factor VIII (FVIII), both essential for thrombotic processes [50]. vWF enters the bloodstream as multimers, and its platelet-binding affinity grows with its molecular weight [51]. Under normal conditions, LSECs do not produce vWF; however, it is found in capillaries in necrotic and congestive liver states. ADAMTS-13, a metalloproteinase featuring a thrombospondin type 1 motif, controls vWF by cleaving its multimers at the Tyr1605 and Met1606 sites within the A2 domain. ADAMTS-13 is predominantly stored in HSCs during normal hemostatic conditions [52]. However, chronic inflammation induces a capillarization of LSECs and a transformation of HSCs into myofibroblasts, which impairs ADAMTS-13 production [53,54].

Additionally, cirrhotic patients have impaired vitamin K production, which results in lower levels of coagulation factors II, V, VII, IX, X, and XI [55]. Despite what appears to be a shift towards an anticoagulated state, these patients actually exhibit elevated levels of factor VIII [56].

In examining the role of factor VIII as a predictor of thrombotic events in cirrhotic patients, Jiang S et al. conducted a study involving 453 patients with esophageal variceal bleeding. The patients were divided into two groups based on the presence of PVT. The study found that factor VIII levels were significantly higher in the group with PVT. This finding identified factor VIII as an independent risk factor for developing PVT within a year of a cirrhosis diagnosis, suggesting its potential use as a predictive marker [57].

The anticoagulant state is also counterbalanced by significantly low levels of anticoagulant proteins such as protein C, protein S, antithrombin, and heparin cofactor II. The role of reduced protein C levels in cirrhotic patients has been extensively studied. In 2013, Tripodi A. et al. demonstrated a pro-coagulant balance due to low protein C levels, which were inversely proportional to the endogenous thrombin potential (ETP) [58]. According to Zhang D. et al., decreased protein C levels are associated with increased D-dimer levels, suggesting an increased risk of PVT in cirrhotic patients [59]. Recently, Zeb F. et al. reported a case of PVT in a young patient without liver fibrosis, highlighting the significant impact of this deficiency on clotting [60]. There has been significant interest in low levels of protein S as a potential predisposing factor for PVT in cirrhotic patients. Specifically, as reported by Hung HC et al. in a study involving a cohort of 349 patients, protein S deficiency alone emerged as the sole independent risk factor for PVT development [61]. Similarly, Zang et al., as previously discussed with regard to protein C, conducted a study involving 188 liver disease patients, suggesting the potential for excluding PVT in the presence of high protein S levels and low D-dimer levels [62].

Regardless of primary and secondary hemostatic factor alterations, cirrhotic patients achieve a delicate hemostatic balance. This condition has been demonstrated by thrombin generation assays, revealing a normal-to-increased thrombin production in these patients [63,64,65]. Specifically, Zanetto A et al. conducted a study involving 108 cirrhotic patients, revealing increased thrombin generation and a pro-thrombotic state in compensated cirrhotic [66].

Moreover, in cirrhotic patients, thrombin generation was found to be more resistant to thrombomodulin, emphasizing a pro-thrombotic tendency [67]. In fact, under physiological conditions, thrombomodulin acts as a cofactor for thrombin during the activation of protein C, leading to the deactivation of activated factors V and VIII, thus playing a role in its anticoagulant function [68].

In the final stages of the coagulation cascade, fibrinogen is transformed into fibrin by thrombin. Later, during fibrinolysis, plasmin breaks down fibrin. Enzymes such as tissue plasminogen activator (t-PA), urokinase activator, and activated factor XII facilitate the conversion of plasminogen into its active form, promoting this biochemical process [69]. Under normal physiological conditions, this mechanism is governed by inhibitors like plasminogen activator inhibitor (PAI), plasmin inhibitor, and thrombin-activatable fibrinolysis inhibitor (TAFI) [69]. In cirrhotic patients, this balance tends towards hyperfibrinolysis due to elevated levels of t-PA and reduced levels of plasmin inhibitor and TAFI [55]. However, there is a simultaneous equilibrium in this mechanism as evidenced by reduced levels of plasminogen and increased levels of PAI.

**Figure 2:**
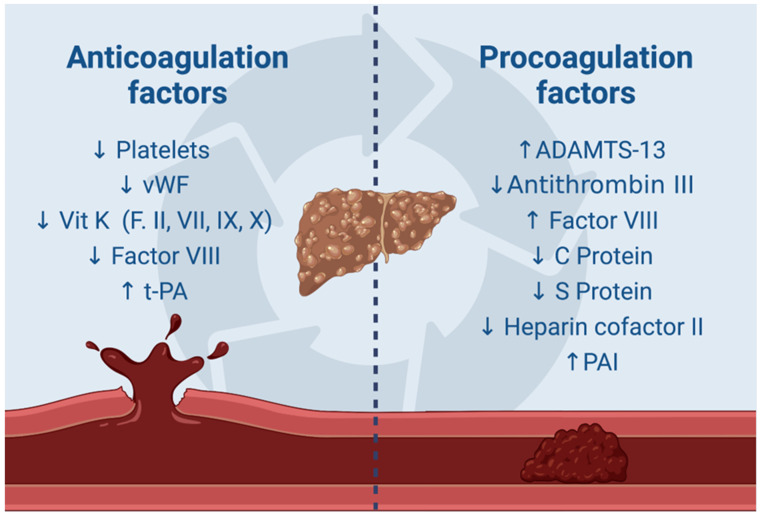
The hemostatic balance in a cirrhotic patient. This image has been generated with Biorender.com (https://biorender.com)—accessed on 14 August 2024.

We propose to synthesize the hemostatic balance of cirrhotic patients through Figure 2 to enhance understanding.

Once it is understood that cirrhotic patients face both anticoagulant and prothrombotic factors due to their disease state, we can delve into how HCC tilts this balance towards a prothrombotic state.

Building upon the molecular perspective already established for cirrhosis, it is noteworthy that the presence of HCC inherently leads to an increase in platelet count. Platelets exhibit a pleiotropic role in HCC, being involved in its progression through the activation of stellate cells, endothelial cells, and pro-tumoral macrophages [70]. Beyond these actions, they also increase the risk of portal thrombosis. As demonstrated by Zanetto A. et al. [71], in cirrhotic patients with HCC, platelets are in a hyperactivated state, evidenced by aggregometry tests using ADP, arachidonic acid (ASPI), and thrombin (TRAP). Additionally, the same study observed a further increase in levels of the platelet adhesive glycoprotein vWF, highlighting not only the impact of thrombocytosis itself but also the enhanced platelet aggregation in disease progression.

Another significant aspect of the pro-thrombotic state in cirrhotic patients with HCC is the increase in plasma fibrinogen. While this finding was already known in the literature and associated with a poor prognosis [72], Zanetto A. et al. further demonstrated not only an increase in fibrinogen levels through thromboelastometry tests but also its correlation with portal thrombosis [73]. Specifically, they found that a maximum clot firmness measured by the FIBTEM (extrinsically activated thromboelastometric test with cytochalasin D) assay exceeding 25 mm was associated with a more than fivefold increased risk of developing portal thrombosis compared to controls. Additionally, in another study, Zanetto A. et al., demonstrated that the pro-thrombotic state in cirrhotic patients with HCC is further exacerbated by the reduced fibrinolysis specifically associated with HCC, independent of cirrhosis status, whether compensated or decompensated [74].

As demonstrated by Poon, R.T et al. in 2003, there is an increase in TF synthesis by hepatoma cells, which was correlated with poor patient prognosis as well as increased cellular and endothelial invasion in HCC [75]. TF is known to be the initiating element of the coagulation cascade, and recent studies have shown that HCC cells frequently exhibit heightened levels of TF on their surface [76,77]. Given that previous studies have linked TF expression to poor patient prognosis in HCC, research should now focus on the interaction between TF-expressing HCC cells and PARs on endothelial cells in the prothrombotic process. This would help determine whether, beyond endothelial invasion, hepatocarcinoma itself can enhance the process of portal thrombosis.

## 4. Molecular Mechanisms of Blood Stasis and Thrombosis in Portal Vein Thrombosis in Cirrhosis

The third component of Virchow’s triad is blood stasis. In the context of a cirrhotic liver, blood stasis occurs due to the formation of regeneration nodules and fibrosis, which alter the hepatic parenchyma. These changes lead to structural modifications in the sinusoids, resulting in blood stasis [78].

Endothelial dysfunction reduces the production of vasodilatory NO while increasing vasoconstrictive factors such as endothelin-1, which worsens blood stasis [79]. This condition causes liver damage and hypoxia, leading to higher VEGF production. VEGF binds to VEGFR-2, triggering a signaling pathway that produces inositol 1,4,5-triphosphate (IP3) and activates phosphatidylinositol 3-kinase (PI3K), which then activates endothelial nitric oxide synthase (eNOS) and promotes the synthesis of NO [80,81].

The release of vasodilators causes dilation of splanchnic arterioles, which leads to a redistribution of blood flow within the portal system [82]. This redistribution results in an enlarged portal vein diameter and the development of portosystemic shunts, reducing portal hypertension and diverting blood away from the liver circulation.

During this phase, HCC heightens thrombotic risk by increasing cellular oxidative damage through the rapid proliferation of HCC cells. This process involves ECM remodeling by activated cells in the tumor microenvironment, such as cancer-associated fibroblasts (CAFs) and type 2 macrophages, which release metalloproteases [39]. This ECM remodeling critically regulates tumor growth, metastasis formation, and invasion into nearby structures [83]. Consequently, in a cirrhotic liver already compromised by altered normal hepatic parenchyma, the proliferation of neoplastic cells leads to reduced cellular vascularization and increased blood stasis. This, in turn, causes an elevation in VEGF and hypoxia-inducible factor 1-alpha (HIF-1α) [39]. The meta-analysis conducted by Cao S. et al. demonstrates that HIF-1α is correlated with greater vascular invasion and, therefore, with the pro-thrombotic risk of HCC [84].

An increase in the portal vein diameter is one of the parameters used to identify patients at risk of developing portal thrombosis in hepatocellular carcinoma. A cutoff of 12.5 mm can aid in recognizing this potential clinical development [85].

A reduction in intraportal flow velocity has been found to independently contribute to the development of PVT, with 15 cm/s being the predictive cutoff velocity for PVT [86,87].

This blood stasis causes shear stress on endothelial cells, initiating a series of molecular mechanisms as previously described, including platelet activation, vWF release, and the secretion of cytokines and chemokines. These events contribute to oxidative stress, express DAMPs, and sustain the prothrombotic process.

In this scenario, while non-selective beta-blockers are known to aid patients with portal cirrhosis by managing portal hypertension, numerous studies and meta-analyses indicate a potential link between this treatment and a heightened risk of portal vein thrombosis, probably due to its hemodynamic effects. Consequently, their role is not entirely clear-cut [88,89].

Up to this point, we have delineated the processes occurring in cirrhosis and hepatocellular carcinoma (HCC) within the framework of Virchow’s triad. Table 1 succinctly synthesizes the additional pro-thrombotic impact of hepatocellular carcinoma.

## 5. Gut–Liver Axis and Portal Vein Thrombosis in Cirrhotic and HCC Livers: The Role of Endotoxemia

It is well established that cirrhotic patients are at a heightened risk of developing systemic infections. This increased susceptibility is attributed to their compromised immune system, the elevated frequency of hospitalizations during disease exacerbations, and the numerous invasive procedures to which they are routinely subjected [90,91,92].

The association between infection and PVT in cirrhotic patients has been prominently observed during the COronaVirus Disease (COVID)-19 pandemic [93,94]. Research by A. Dalbeni et al. has challenged the conventional belief that decompensated cirrhosis is the predominant cause of PVT, emphasizing a higher incidence of PVT in non-decompensated cirrhotic patients [95]. The authors suggest that other factors, like infections, could contribute to PVT, pinpointing bacterial infections, both Gram-negative and Gram-positive, as independent risk factors. In this context, research has concentrated on examining the microbiota of cirrhotic patients and its contribution to the development of PVT. Huang X. et al. performed a comparative study on the gut microbiota composition and its potential role in PVT development, involving twelve patients with liver cirrhosis and PVT, as well as a control group of twenty-one patients without PVT [96]. The research also explored potential relationships between variations in gut microbiota composition and coagulation parameters, such as platelet count, prothrombin time, international normalized ratio, fibrinogen, and D-dimer. Elevated levels of platelets and D-dimer were observed in cirrhotic patients with PVT compared to the control group. The study identified a significant decrease in Bacteroides’ levels in the PVT group, suggesting a potential involvement in PVT development. To test this hypothesis, Bacteroides were introduced into a CCL4-induced cirrhosis model, and ultrasound was used to confirm PVT formation. The addition of Bacteroides resulted in a noticeable reduction in PVT volume and improved blood flow. Additionally, the elevated levels of fibrinogen, D-dimer, and P-selectin observed in the CCL4 group decreased following Bacteroides’ supplementation [96].

Considering the risk of portal vein thrombosis in cirrhotic patients during bacterial infections, it is crucial to note the impact of endotoxemia. In these patients, immune system dysfunction and impaired Kupffer cell function, which normally clears endotoxins, are significant factors [97]. It is evident that in cirrhotic patients, as Kupffer cell function diminishes, there is an increase in endotoxins, leading to a pro-thrombotic environment. This occurs through heightened tissue factor production, which activates the coagulation cascade, and increased expression of adhesion molecules, promoting thrombus formation [98]. At the same time, disturbances in the integrity of the intestinal mucosa and imbalance in the gut microbiota maintain systemic inflammation, promoting the movement of bacteria and their pathogen-associated molecular patterns (PAMPs) into circulation [99,100]. Among these factors, lipopolysaccharide (LPS) plays an important role in portal thrombosis [101].

In particular, circulating LPS interacts with Toll-like receptors (TLRs) found on endothelial cells, leading to their activation and subsequent release of vWF and factor VII [102]. Research by Jäckel S et al. has demonstrated that LPS can also bind to TLR2 on hepatic endothelial cells, promoting the expression of the vWF precursor [103]. Additionally, LPS influences the vWF-FVIII system by inducing the secretion of Weibel–Palade bodies in endothelial cells via TLR4, as reported by Carnevale R. et al. [104]. Considering that TLR2 and TLR4 are widely distributed on the surface of platelets, LPS is also involved in platelet activation. Platelet activation is triggered by LPS binding, resulting in the release of α-granules and dense granules [105]. These granules interact with vascular endothelial cells and promote platelet-dependent thrombosis. The activation of platelets leads to the release of P-selectin, facilitating platelet–monocyte aggregation, the release of inflammatory molecules, and endothelial cell adhesion, all contributing to thrombus formation [106,107]. Understanding the complex interactions between immune dysfunction, changes in gut microbiota, and vascular inflammation is essential for comprehending the development of portal vein thrombosis in cirrhotic patients.

**Figure 3:**
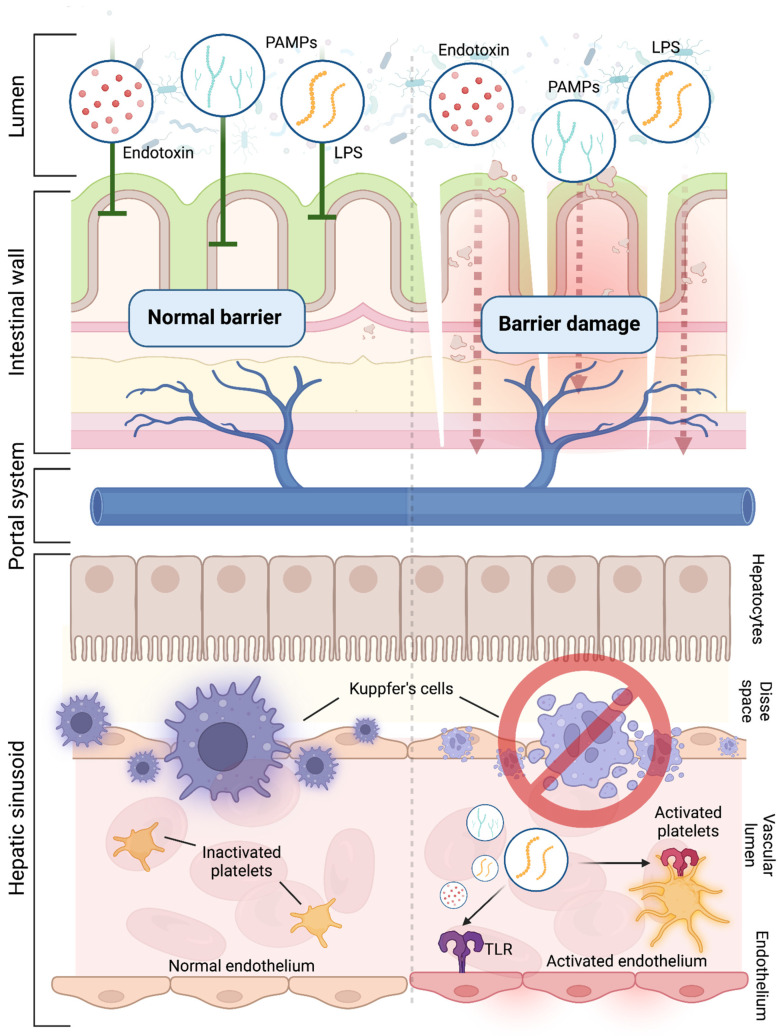
The role of endotoxemia on portal vein thrombosis in cirrhotic livers. This image has been generated with Biorender.com (https://biorender.com)—accessed on 14 August 2024.

Currently, there is a lack of specific studies linking intestinal microbiota to portal vein thrombosis in patients with liver disease. Therefore, it remains unclear whether HCC itself, along with its tumor microenvironment, is solely responsible for the development of portal vein thrombosis. However, it is important to consider that several studies have observed elevated levels of Bacteroidetes in the HCC microenvironment [108,109,110,111,112,113]. This suggests a potential broader impact of endotoxemia related to dysbiosis caused by liver pathology, whether tumorous or not, on the heightened risk of portal vein thrombosis (Figure 3).

## 6. Molecular Mechanisms of Microvesicles in Portal Vein Thrombosis in Cirrhotic Patients

In the literature, researchers are investigating whether cirrhotic patients’ heightened prothrombotic risk is associated with increased levels of microvesicles released into circulation by platelets, endothelial cells, and leukocytes compared to those found in healthy individuals. These microvesicles play a vital role in cellular communication by transferring surface ligands through receptors to target cells and releasing functional proteins, transcription factors, and genes [114,115,116]. As observed in other pathologies, platelet-released microparticles exhibit prothrombotic activity [117,118]. To date, a correlation has been observed between these microvesicles and clinical decline and unfavorable results in patients with cirrhosis [119,120,121]. Among these microvesicles, those expressing phosphatidylserine have shown heightened prothrombotic effects by serving as platforms for assembling coagulation factors such as factor Xa and prothrombinase complexes. The studies of Wu et al. have shown that endothelial cells exposed to cirrhotic serum exhibit increased phosphatidylserine exposure compared to those exposed to healthy serum, which supports shortened coagulation times and increased FXa, thrombin, and fibrin formation [122]. Additionally, Campello et al. discovered, in a cohort of HCV-related cirrhotic patients, that extracellular vesicles from platelets expressing CD61 heighten the risk of portal vein thrombosis [123]. Microvesicles play significant roles in the pathogenesis of portal vein thrombosis in cirrhotic patients due to their procoagulant, inflammatory, and proangiogenic properties. Understanding these molecular mechanisms is crucial for developing targeted therapies to enhance clinical outcomes in this patient population.

Regarding HCC cells, there is evidence that they release microvesicles originating from endothelial cells, platelets, and leukocytes, which express TF or thrombomodulin [124,125,126]. However, these studies have not shown a direct role in thrombogenesis, instead concentrating on identifying tumor cells in comparison to cirrhotic controls without HCC. Similarly, while the release of exosomes has been linked to tumorigenesis and metastasis, it has not been associated with portal vein thrombosis [127]. Another notable characteristic of HCC cells is their formation of NETs, yet there is no evidence of their direct role in thrombogenesis. NETs have been observed in hepatic cells of mice with metabolic dysfunction-associated steatohepatitis (MASH), where they contribute to HCC progression [128]. Given the role of NETs studied in cirrhotic patients, as described in the section on endothelial dysfunction, it can be speculated that HCC contributes to thrombogenesis by increasing NETs through HCC cells.

## 7. A Direct Role of HCC Cells in Portal Vein Thrombosis: Molecular Mechanisms

Having explored additional pro-thrombotic mechanisms of HCC in cirrhotic livers, we now delve into a distinctive feature of HCC cells. The tumor microenvironment of HCC comprises a variety of cells, including cancer stem cells (CSCs). Numerous studies have highlighted the presence of CSCs in tissues affected by portal vein thrombosis. In Abdelgawad IA’s 2020 study [129], CSCs expressing the epithelial cell adhesion molecule (EpCAM) were exclusively identified in patients with HCC. This finding linked these cells to both PVT and tumor metastasis. In 2015, the meta-analysis by Zhong C. et al. [130] determined that CD133-expressing CSCs are primarily associated with PVT, whether considered as an independent condition or as part of vascular invasion. Notably, there was no observed correlation with cirrhosis, which is typically a pro-thrombotic characteristic of HCC.

The molecular mechanism underlying CSCs in PVT remains incompletely understood; Zhang et al. proposed an RNA-mediated protein mechanism resulting in IL-6 transcription, stimulating metastasis and tumor progression, and potentially increasing PVT [131]. Similarly, Guo W. et al. discovered that a non-coding RNA associated with ICAM-1 not only was involved in ICAM-1 expression on HCC CSCs but was more prevalent in the presence of portal vein thrombosis [132]. Further studies are crucial to fully understand the molecular mechanisms involved.

Another subgroup of tumor cells studied within the tumor microenvironment includes circulating tumor cells, which seem to derive from ECM transformation in EMT [133]. However, the specific molecular mechanisms underlying PVT for these cells have yet to be described. Nonetheless, given their circulation within the capillary bed, they may correlate with PVT development.

Wang T. et al. provided an alternative view on the formation of PVT in HCC, showing that it can originate from circulating HCC cells. They identified a cell line named CSQT-2 derived from HCC, and when this cell line was introduced into mice, it resulted in the development of portal vein thrombosis [134]. Ongoing research aims to establish whether the cells causing PVT arise from the same cell line as the primary HCC or if they undergo clonal changes within the tumor microenvironment.

Guo W. et al. indicated different origins between PVT cells and the associated tumor [135].

While we have identified specific differentially expressed genes such as CD24 and EpCAM for CSCs and hypoxia-induced factor 1 (HIF1)-a [84], another major line of research focuses on the role of epigenetic mutations in HCC cells. Specifically, studies noted an association between decreased DNA methylation and the vascular invasion of HCC, linked to the overexpression of target genes tumor necrosis factor receptor superfamily member 10A (TNFRSF10A) and family with sequence similarity 83 member D (FAM83D), respectively [136,137]. Conversely, Xu F. et al. found similar outcomes when investigating increased DNA methylation levels and the expression of the target gene SCAN domain containing 3 (SCAND3) [138]. At the same time, molecular research was conducted on micro ribonucleic acid (miRNA), observing in particular how through the downregulation of miRNA-381, the expression of VEGFA occurs, which we have seen to be involved in angiogenesis as well as in the promotion of portal venous thrombosis [139]. Likewise, the epigenetic role of the long non-coding RNA (lncRNA) has been linked to vascular invasion in HCC, contributing to its pro-thrombotic effects by activating other target genes such as cyclin-dependent kinase 5 (CDK5), FOS like antigen 2 (FOSL2), and CD44v6 [140,141]. A schematic summary is available in Table 2.

## 8. The Role of Thrombophilia in the Genesis of Portal Vein Thrombosis

Patients with cirrhosis and HCC continue to face a persistent risk of thrombosis despite maintaining a delicate hemostatic balance. It is crucial to pinpoint specific markers that can accurately identify which of these individuals are at increased risk of developing PVT. Current research is actively investigating the potential impact of thrombophilic mutations in this context, yet findings remain inconclusive at present. Specifically, Mangia et al. found no statistically significant difference in thrombophilia prevalence between cirrhotic patients with PVT and those without it [96]; Delgado et al. reported a similar finding [104]. Additionally, in 1997, Mahmoud et al. conducted a retrospective analysis and identified Factor V Leiden (FVL) in 3.1% of their 32 patients. However, they did not find a definitive link between FVL and the development of PVT [105].

Cagin et al. noted a comparable link between FVL and PVT, though they did not establish a direct connection [106]. This pro-thrombotic mutation, however, appeared more frequently in cirrhotic patients with PVT.

The significance of the prothrombin G20210A (PTHR) and Methylenetetrahydrofolate Reductase (MTHFR) mutations in diagnosing PVT in cirrhotic patients varies [107,108,109]. The association of MTHFR mutations with PVT risk has been supported by studies conducted by Gabr et al. in Egypt and Pasta et al. in Italy [110,111].

Due to the significant number of studies that are retrospective in nature, the role of thrombophilic mutations in the development of portal vein thrombosis in cirrhotic patients has not been fully clarified.

Despite the presence of antiphospholipid antibodies reported in cirrhotic patients, with prevalence directly influenced by the severity of liver disease, clarity regarding their role in PVT remains elusive [112].

On the contrary, the role of fibrinolysis in this context has been substantiated by several studies, highlighting that elevated TAFI levels are associated with an increased risk of thrombosis [113,114]. Conversely, PAI-1, t-PA, plasminogen, and α2-antiplasmin did not yield conclusive results [115,116,117].

Considerable focus has been directed towards investigating various coagulation factors. Some studies previously explored the association between Factor VIII and Protein C, revealing no correlation with PVT but indicating a stronger connection with fibrosis progression [118,119,120]. Similarly, research into the ratio of Factor II and Protein C did not produce favorable results [118,120]. On the flip side, diminished levels of Protein C and antithrombin were associated with PVT, along with the ratio of thrombin–antithrombin complex (TAT) to tissue plasminogen activator–inhibitor complex (tPAIC) [121].

All previous studies mentioned so far do not specify a greater pro-coagulant tendency in cirrhotic patients with HCC compared to cirrhotic patients without HCC. Further research is needed not only to understand the true impact of thrombophilia on splanchnic venous thrombosis but also to assess its specific relationship with HCC.

## 9. Conclusions

Given the clinical impact of portal vein thrombosis on the prognosis of cirrhotic patients with HCC, several research avenues are being pursued with the aim of developing specific scores or identifying parameters capable of identifying patients at higher risk of developing this complication, with the ultimate goal of preventing such an event.

Considering the unreliability of standard laboratory hemocoagulation tests for cirrhotic patients, Lewis CS et al. conducted a study demonstrating how the incorporation of Factor V and Protein C levels enhances the Model for End-Stage Liver Disease (MELD) and contributes to evaluating the risk of PVT [142].

Recent progress in predicting PVT in cirrhotic patients has been underscored in the literature, particularly through the validated calculator developed by Nie GL et al. [143].

It is noteworthy that the calculator’s significant impact was influenced by both clinical factors and numerical variables such as platelet count, D-dimer, portal vein diameter, and portal vein velocity. This underscores that thrombophilia and coagulation per se are just some of the many factors contributing to the development of PVT in cirrhotic patients. Hence, this clinical condition appears to be far more complex than currently understood.

Once again, previous scores are calibrated for cirrhotic patients, but new evidence is also emerging concerning HCC. Recently, Fukui S. et al. reported findings showing that analyzing clot waveforms using activated partial thromboplastin time (APTT), TF, and factor IXa reveals a pro-thrombotic trend in cirrhotic patients with HCC [144]. Sherman CB et al. implemented the A-VENA Criteria, which involves AFP levels (>1000 ng/dL) and portal vein thrombosis imaging characteristics (venous expansion, enhancement of the thrombus, neovascularization, adjacency to HCC), demonstrating accurate identification of portal vein thrombosis [15]. Li T. et al. showed how Protein induced by vitamin K absence or antagonist II (PIVKA-II) levels can be used in diagnosing portal vein thrombosis in HCC patients [145]. The aim is always to identify HCC patients at higher thrombotic risk, but there is another facet: using these markers as potential targets for therapy to ensure improved clinical outcomes.

In fact, returning to the evidence regarding CSCs expressing CD24 and EpCAM and their association with PVT in HCC, researchers including Tong M. et al. have shown that the use of neutralizing monoclonal antibodies against Annexin A3 (ANXA3) effectively eliminated these cells. This inhibition not only suppressed HCC growth but also demonstrated considerable potential in the prevention and treatment of portal vein tumor thrombosis [146].

Therefore, various research avenues are currently being pursued, and numerous additional studies are certainly needed to establish, on a large scale, scores or parameters that can both identify HCC patients at higher risk of developing portal vein thrombosis and highlight target markers to enhance therapeutic response.

## Figures and Tables

**Table 1:** The impact of hepatocellular carcinoma (HCC) in portal vein thrombosis in cirrhotic liver.

Factor	Role in Portal Vein Thrombosis in HCC	References
Endothelial Damage and Inflammation	HCC cells secrete IL-6 and TNF-α, contributing to endothelial damage and increasing susceptibility to thrombosis.	[34]
Endothelial-to-Mesenchymal Transition (EMT)	HCC induces EMT in vascular endothelial cells via the Cyclin G1/PI3K/Akt/GSK-3/Snail pathway, promoting portal thrombosis.	[35,36]
Platelet Activation	HCC increases platelet count and induces hyperactivation, enhancing platelet aggregation and thrombotic risk.	[65,66]
Fibrinogen levels	Elevated fibrinogen levels in HCC patients correlate with increased risk of portal thrombosis.	[67,68]
Fibrinolysis Impairment	HCC decreases fibrinolysis, further exacerbating the pro-thrombotic state.	[69]
Tissue Factor (TF) Expression	HCC cells increase TF synthesis, initiating the coagulation cascade and promoting thrombosis.	[70,71,72]
ECM Remodeling and Hypoxia	HCC-related ECM remodeling and hypoxia increase VEGF and HIF-1α, correlating with greater vascular invasion and thrombotic risk.	[34,78,79]

**Table 2:** Genetic and epigenetic mechanisms of HCC in the genesis of portal vein thrombosis.

Study	Results	Mechanism/Cell Type
Abdelgawad IA et al. [122]	EpCAM-expressing CSCs exclusively in HCC patients linked to portal vein thrombosis and metastasis.	CSCs
Zhong C. et al. [123]	CD133-expressing CSCs associated with portal vein thrombosis, independent of cirrhosis.	CSCs
Zhang et al. et al. [124]	RNA-mediated IL-6 transcription in CSCs stimulating metastasis and portal vein thrombosis.	CSCs
Guo W. et al. [125]	Non-coding RNA linked to ICAM-1 expression in HCC CSCs and portal vein thrombosis.	CSCs
Wang T. et al. [127]	Portal vein thrombosis originating from circulating HCC cells (CSQT-2 cell line).	Circulating tumor cells
Fan X et al. [129] Liao W et al. [130] Xu F et al. [131]	DNA methylation changes (TNFRSF10A, FAM83D, SCAND3) and vascular invasion in HCC.	Epigenetics
Wang J. et al. [132]	miR-381 downregulation and VEGFA expression promoting angiogenesis and portal vein thrombosis.	miRNA
Li M. et al. [133] Song LN et al. [134]	lncRNA role in HCC vascular invasion via CDK5, FOSL2, and CD44v6 activation.	lncRNA
